# Measuring Physical Properties of Electrospun Nanofiber Mats for Different Biomedical Applications

**DOI:** 10.3390/membranes13050488

**Published:** 2023-04-30

**Authors:** Sarah Vanessa Langwald, Andrea Ehrmann, Lilia Sabantina

**Affiliations:** 1 Faculty of Engineering and Mathematics, Bielefeld University of Applied Sciences and Arts, 33619 Bielefeld, Germany; sarah_vanessa.homburg@hsbi.de; 2 Faculty of Clothing Technology and Garment Engineering, School of Culture + Design, HTW Berlin—University of Applied Sciences, 12459 Berlin, Germany

**Keywords:** ImageJ, apparent density, porometer, scanning electron microscopy (SEM), specific surface area, fast Fourier transform (FFT), water contact angle, surface roughness, tensile test, conductivity

## Abstract

Electrospun nanofiber mats are nowadays often used for biotechnological and biomedical applications, such as wound healing or tissue engineering. While most studies concentrate on their chemical and biochemical properties, the physical properties are often measured without long explanations regarding the chosen methods. Here, we give an overview of typical measurements of topological features such as porosity, pore size, fiber diameter and orientation, hydrophobic/hydrophilic properties and water uptake, mechanical and electrical properties as well as water vapor and air permeability. Besides describing typically used methods with potential modifications, we suggest some low-cost methods as alternatives in cases where special equipment is not available.

## 1. Introduction

Electrospinning allows for producing nanofiber mats from diverse polymers or polymer blends, including various nanoparticles, and in this way tailoring the nanofiber materials in a broad range [1,2,3]. Their large specific surfaces as well as other physical and chemical properties make such nanofiber mats highly suitable for biotechnological and biomedical applications, such as wound healing or tissue engineering [4,5,6].

Naturally, nanofiber mats for biomedical applications need special properties, especially being not cytotoxic, but depending on the exact application, they can be desired to be biodegradable or waterproof, have antibacterial of fungicide properties, etc. [7,8,9,10]. However, their morphological, mechanical and other physical properties may also be important for the planned application, although these values are often less intensively investigated than chemical and biochemical properties and often only briefly described in the methodic sections. Nevertheless, the mechanical properties are decisive for the lifetime of a nanofibrous product and the limits of its potential application, while cell adhesion depends on morphological parameters, hydrophobicity and water uptake are among the parameters controlling liquid transport, which is important for wound dressing, and porosity and water vapor/air permeability are physical parameters influencing the filtration of liquids or gases, respectively. The porosity is often mentioned as an important parameter for wound exudate transport and cell adhesion [11,12,13,14,15,16,17]. While the porosity describes the amount of porous volume inside the nanofiber mat volume, the pore size distribution is also often taken into account [14,15,16,18,19]. Other morphological parameters are the nanofiber diameters [16,20,21,22,23,24] and their orientation [25,26,27] as well as the surface roughness and nanofiber mat thickness [28]. Besides such structural features, the hydrophobic/hydrophilic properties of nanofiber mats [11,16] and their water uptake [17,24,29] are often reported. Other often-mentioned parameters are mechanical [11,15,17,18,19,20,21,23,24] and electrical properties [18,30] as well as water vapor and air permeability [31,32]. This review gives an overview of the different measurement methods for these parameters, discusses differences in the gained results and suggests some less well-known inexpensive alternatives to the typically used instruments that are not always available for each study.

## 2. Porosity

The porosity describes the volume of voids inside a given volume of a nanofiber mat. Firstly, it must be mentioned that there can be open as well as closed pores, the latter of which are not accessible for all methods described below [33,34,35]. However, for typical nanofiber mats, pores can be expected to be openly accessible to any test fluid, so that for most nanofibrous membranes, no differences between the measurement principles are expected, whether they take into account closed pores or not.

One of the methods that would also measure closed pores is the Archimedean principle [36]. Pati et al. used a specific gravity bottle filled with ethanol in which the nanofibrous scaffold was dipped and afterwards removed again [37]. The porosity was then calculated according to

(1) $$Porosity=\frac{\left(m_{2}-m_{3}-m_{S}\right)}{m_{1}-m_{3}}\times 100\%$$

with the mass $m_{1}$ of the specific gravity bottle filled with ethanol, the mass $m_{2}$ of the bottle with ethanol and the scaffold, the mass $m_{3}$ of the bottle after taking out the scaffold again, and $m_{S}$ the mass of the scaffold. Dividing both numerator and denominator by the density of ethanol, it is observable that the porosity is determined as the volume of the ethanol taken out of the bottle with the scaffold, i.e., of the ethanol that was sticking in its pores, divided by the volume of the scaffold with ethanol. Safari et al. used the same principle based on deionized water in which their nanofiber mats were immersed for 15 min, taken out, quickly dried at the sample surface and weighed, so that the porosity could be calculated as the mass of the uptaken water, divided by the sample mass [38].

Without directly using the Archimedean principle, Kahdim et al. calculated the porosity by soaking their nanofiber mats in phosphate-buffered saline (PBS) solution for 24 h, measuring the sample mass before and after PBS uptake and calculating the porosity according to this fluid uptake and the PBS density [39]. Here, it is not mentioned whether the samples were also dried on both surfaces before weighing them. Using immersion of the dried nanofiber mat in n-butanol for 2 h, Wang et al. as well as Chen et al. calculated the porosity from the densities of membrane and n-butanol as well as the measured dry and wet mass of the nanofiber mat [40,41].

Other research groups used a similar technique of wetting a sample in a fluid, but measured the volumes of the fluid instead of the immersed nanofibrous mat. Salehi et al. calculated the porosity of poly(*ε*-caprolactone)(PCL)/gelatin nanofiber mats by immersion in ethanol and calculated the porosity as

(2) $$Porosity=\frac{V1-V3}{V2-V3}\times 100\%$$

with the initial volume V1 of ethanol, the volume V2 after immersion of the nanofiber mat and the volume V3 of the ethanol without the soaked mat, taken out after 10 min [42]. Ghaee et al. also used ethanol to investigate the porosity of their PCL nanofiber mats by this liquid displacement technique [43], similarly to Esmaeili et al. for cellulose acetate/polyurethane nanofiber mats [44], while Chen et al. used the same method for their poly(lactic acid) (PLA)/regenerative cellulose composite scaffolds with hexane instead of ethanol [45].

Several papers mention a gas pycnometer as a possibility to measure the volume of a porous sample which enables calculating the theoretical density of a sample and correspondingly the porosity by the formula

(3) $$Porosity=\left(1-\frac{\rho_{exp}}{\rho_{theo}}\right)\times 100\%$$

with the measured density $\rho_{exp}$ and the theoretical density $\rho_{theo}$ of the material under investigation [46]. The easiest form of a gas pycnometer contains two chambers, one of them with well-known reference volume, while the sample is introduced into the other one. A measuring gas is introduced into one of the chambers and allowed to expand into the second chamber through a valve. The sample volume can then be calculated from the previously known volumes of the empty sample chamber and the reference chamber as well as the pressure of the firstly filled chamber and the equilibrium pressure after gas expansion [47]. While this method thus necessitates more sophisticated equipment than the previously described methods based on fluids filling the pores of the nanofiber mat, the latter take more time and are more error prone, especially when the experimental procedure is not perfectly described, e.g., regarding drying the sample surfaces after dipping or not.

Equation (3) can also be used for other ways to determine the apparent density of a sample, in the easiest way by measuring its mass as well as its thickness and lateral dimensions, where the error range is mostly influenced by the thickness measurement, which will be discussed in Section 8. Nevertheless, this relatively simple method can be used to give an estimate of the porosity, keeping in mind that irregularities of the sample thickness and its compressibility will potentially cause deviations from the real value. Porosity calculations by the apparent density, calculated from the sample volume, were reported by several research groups for different nanofiber mat materials, such as polyamide-6/polyvinylpyrrolidone [48], polyurethane [49] or collagen-coated poly(l-lactic acid)-*co*-poly(*ε*-caprolactone) [50].

Besides these methods, which are used to determine 3D pore structures, some papers also mention calculating the surface porosity, typically based on scanning electron microscope (SEM) images and their evaluation by ImageJ (National Institute of Health, Bethesda, MD, USA) or partly automated by the plugin DiameterJ [49,51,52,53].

Finally, a direct measurement of the porosity is enabled by laser metrology, measuring the surface of an electrospun nanofiber mat on the collector, followed by completely densifying via heat treatment and afterwards measuring the surface profile again, so that the porosity can be calculated from the vertical shrinkage [54,55].

## 3. Pore Size Distribution

While the porosity describes the overall volume of the pores in a given sample, the pore size distribution is sometimes even more important in biomedical scaffolds since it defines which pores are available for cells or can release a drug. In the easiest way, pore sizes are measured on the surface or along cross-sections of samples, typically from SEM images. Agueda et al. describe that they used ImageJ to investigate pore sizes from 3 areas per sample from SEM images taken with magnification of 2000× and 5000×, measuring 30 pores per sample [56]. Liu et al. similarly examined pore sizes from SEM images of their nanofiber mats, taken with magnifications of 5000× and 20,000×, averaging over 100 pore areas [57]. Tahami et al. also used ImageJ to measure pore sizes in SEM images, while not exactly describing the number of measurements [58], while Stella et al. showed histograms of the pore size distributions, which in principle allow for counting the number of measurements per sample, again taken with ImageJ from SEM images [59].

**Figure 1:**
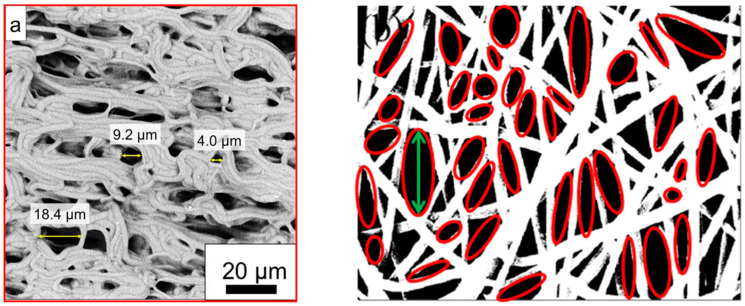
Scanning electron microscope (SEM) images of nanofiber mats for pore size determination: (**a**) covalently crosslinked poly(ε-caprolactone) at a temperature of 60 °C, from [58], originally published under a CC-BY license; (**b**) polyethylene terephthalate/polycaprolactone blends. From [53], copyright (2020), with permission from Elsevier.

Only a few groups describe how they defined the pore size that they measured. Zhang et al. described measuring a reversible change in pore size by analyzing 30 pores per sample with ImageJ in their SEM images by measuring the longest diameter, as shown in Figure 1a [60]. Havlícek et al., on the other hand, used Matlab to determine the pore sizes as equivalent circle diameters, i.e., they measured the pore areas and calculated the diameter of a circle with identical area [61]. Krysiak et al. similarly fitted ellipses to the pores in the SEM images by ImageJ and calculated their areas [62]. Nejad et al. also worked with ellipses fitted into the pores (Figure 1b), but gave the larger diameter as the pore size [53]. In some papers, the average pore size could be estimated from SEM images, without a detailed explanation of how this value was determined [63].

Due to the broad range of possibilities to define the pore size, it is strongly suggested to always clearly mention the chosen definition as well as the number of investigated pores in a paper.

Besides these 2D methods, some groups chose 3D pore measurement methods. One of them is the Barrett–Joyner–Halenda (BJH) technique, allowing analysis of pores between 1.7 nm and 300 nm [64]. This method is based on N_2_ adsorption–desorption isotherms, taken at liquid nitrogen temperature [65,66,67,68], i.e., similarly to the Brunauer–Emmett–Teller (BET) surface area measurements described in the next section. Generally, the BJH method as well as further developments are based on measuring the film formation on the mesopore walls in dependence of the condensation pressure, taking into account the so-called Kelvin-type relation describing capillary condensation, meaning that mesopores covered with an absorbed fill will instantaneously be filled [68]. In particular, the extended BJH-KJS (Kruk-Jaroniec and Sayari) method was found to allow for accurately calculating mesopore volumes [69].

A capillary flow porometer can also be used to investigate the pore sizes of nanofiber mats [41,70]. In this method, the sample pores are filled with a wetting liquid that is afterwards blown out of the pores by a pressurized gas or liquid [71], where smaller pores need a higher pressure to be emptied, i.e., the measured flow rate depends on the proportion of filled pores that block the flow so that there is zero flow at low pressure, while at a certain high pressure, all pores are emptied, and the flow rate becomes identical to the value measured for the dry sample at the same pressure [72]. It should be mentioned that this method may depend on the wetting fluid [73,74] and the used instrument [75], and thus, the results should be compared with other methods to evaluate their reliability.

Generally, some other methods are available, although less often reported in the recent literature, to evaluate the pore size distribution of nanofiber mats, such as mercury intrusion porosimetry [76]. Another method that is less well-known but often more readily available than a porometer or nitrogen absorption techniques, is thermoporometry, also known as thermoporosimetry or cryoporometry [77,78,79,80]. This calorimetric method is based on the melting or freezing point depression of the pore liquid, which can be measured with a laboratory differential scanning calorimetry (DSC) instrument by fast cooling the sample wetted with deionized water to −30 °C or lower and then slowly (e.g., with a heating rate of 0.1–1 K/min) heating it up to a temperature slightly above 0 °C [81,82]. A summary of the theory behind the technique can be found elsewhere [83]. Although the DSC results are less straightforward to interpret than the results of other techniques [84], some authors report thermoporometry measurements of nanofiber mats.

Abolhasani et al. used thermoporometry to measure the porosity of poly(vinylidene fluoride-trifluoroethylene) (P(VDF-TrFE)) nanofiber mats [85]. Gustafsson et al. compared dry- and wet-state porometry methods for the analysis of virus removal filter paper [86]. They found that thermoporometry by DSC is particularly useful to characterize the pore-size distribution in the wet state. Fashandi et al. mentioned that their originally hydrophobic polystyrene nanofiber mat was hydrophilized by oxygen plasma treatment to enable thermoporometric measurements, showing a pore size radius distribution around 20–50 nm [87]. As these few examples show, characterization of the pore size distribution of nanofiber mats is also possible by using a DSC instrument, which is more often available in laboratories than more-specialized porometers, etc.; however, care should always be taken when comparing the results gained with different methods, as the simple comparison of different pore size definitions in 2D optical methods already showed.

## 4. Specific Surface Area

Among the very special properties of nanofiber mats is their large specific surface area. While this value is often mentioned as a reason why nanofiber mats are especially useful for a certain application, its value is scarcely measured. The most common measurement technique is based on the aforementioned BET adsorption–desorption isotherms of N_2_ gas on the sample surface [68,88,89,90,91,92,93,94]. Generally, a wider hysteresis loop in the adsorption–desorption curve indicates a more mesoporous structure of the nanofiber mat [68].

**Figure 2:**
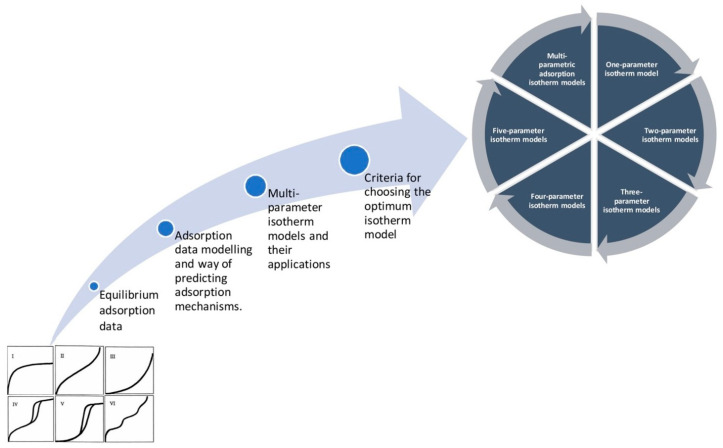
Estimation of isotherm model from equilibrium adsorption data. From [98], copyright (2020), with permission from Elsevier.

To evaluate these curves, it is necessary to differentiate between the different adsorption isotherms. Many nanofiber mats reported in the literature belong to type IV [95], while type I or a pressure-dependent change between these types are also found [96,97]. Differentiation between the different types of adsorption isotherms is possible by fitting a range of possible equations to the measured curves, while a first idea of the type can already be gained by looking at the slopes of the measured adsorption–desorption curves (Figure 2) [98]. For a comprehensive overview of mono- and multi-parametric isotherm models with the corresponding regression equations, the reader is referred to the review paper of Al-Ghouti and Da’ana [98].

**Figure 3:**
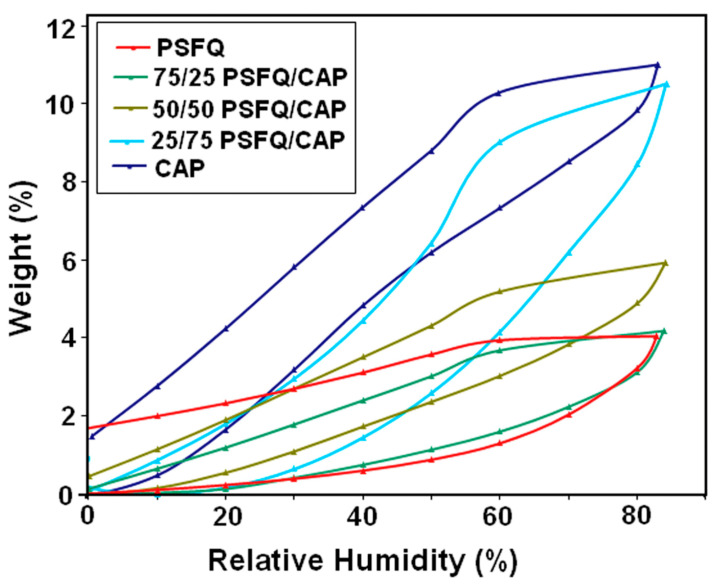
Moisture sorption isotherms for different quaternized polysulfone-based blends with tunable cellulose acetate phthalate content. From [100], copyright (2018), with permission from Elsevier.

While the BET method is based on N_2_ adsorption–desorption curves, it is also possible to use the water vapor sorption capacity with a gravimetric analyzer [99]. This dynamic measurement technique by an automated gravimetric analyzer is based on an ultrasensitive micro-balance, measuring the mass change of a sample while the humidity in the sample chamber is increased from less than 1 to 90% in steps of 10%, where the sample is allowed to reach equilibrium for 10–20 min per step [100]. Similarly, desorption curves were measured during decreasing relative humidity. The results, depicted in Figure 3, show hysteresis curves, but are partly not closed, as opposed to the N_2_ isotherm curves visible in Figure 2. These curves were interpreted as type IV, and average pore sizes around 1 nm were calculated from them [100].

While the specific surface area is one of the parameters that need special equipment to being measured, the diameters and orientations of nanofibers in an electrospun membrane are usually measured from SEM images, as discussed in the next sections.

## 5. Nanofiber Diameter

**Figure 4:**
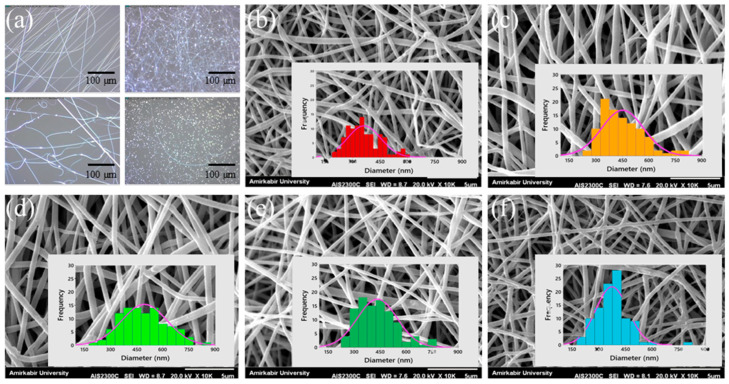
(**a**) Digital microscope images for pre-assessing the electrospinning conditions (scale bar: 100 μm, 370×), SEM images (scale bar: 5 μm) and nanofiber diameter histograms of (**b**) polyethylene terephthalate (PET), (**c**) PET/polycaprolactone (PCL) (3:1), (**d**) PET/PCL (1:1), (**e**) PET/PCL (1:3), (**f**) PCL. From [53], copyright (2020), with permission from Elsevier.

The diameters of nanofibers in an electrospun membrane are usually obtained from SEM images and either given as average with standard deviation or as distribution, sometimes as distribution boxplots [101], but mostly as a histogram. In the latter case, typically 100 or more fiber diameters per sample are measured to prepare a histogram [39,41,48,49,90], as shown in Figure 4 [53]. In most cases, the diameters are measured manually by ImageJ [14,43,56,57,68], while a few groups mention other software [41,44,90] or do not mention the software used [70]. Only a few papers mention the use of the ImageJ plugin DiameterJ or Super Pixel, which can in principle be used to automatically measure fiber diameters from SEM images [51,102,103,104], possibly because of problems with this automatic fiber detection caused by partial fibers or fiber intersections with dark spots [105]. On the other hand, a few groups suggested their own image analysis tools for this purpose [106,107].

## 6. Nanofiber Orientation

**Figure 5:**
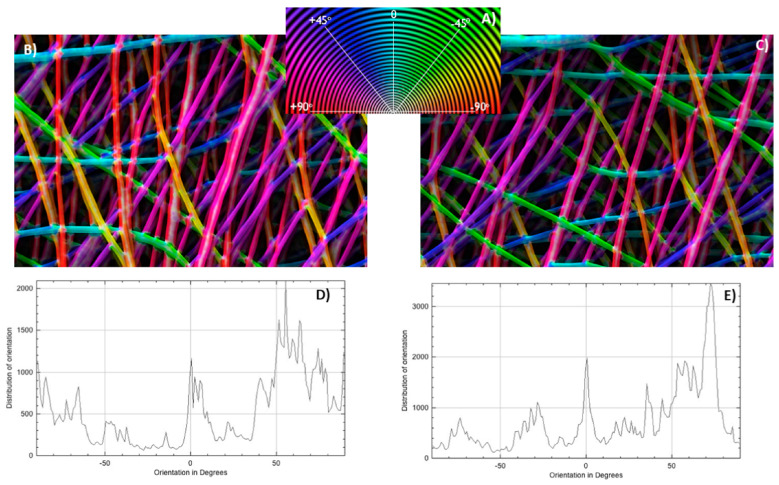
OrientationJ results: (**A**) circular color map coding; (**B**) color map image of batch 1, electrospun with 262 rad/s mandrel rotation speed; (**C**) color map image of batch 8, electrospun with 183 rad/s; (**D**) trace of orientation of batch 1; (**E**) trace of orientation of batch 8. From [105], copyright (2020), with permission from Elsevier.

Oriented nanofibers can be produced, e.g., by a fast rotating collector cylinder. Similarly to the nanofiber diameter distribution, the orientation of the nanofibers in an electrospun membrane is also usually determined from SEM images. The fiber orientations can be measured manually in ImageJ [108] or other software [109]. An interesting possibility to automatically detect fiber orientations is given by the ImageJ plugin OrientationJ, as depicted in Figure 5 [105]. While the color-coded fiber images (Figure 5B,C) enable checking the correctness of the detected orientation, the orientation graphs show the quantitative evaluation of the fiber orientation.

**Figure 6:**
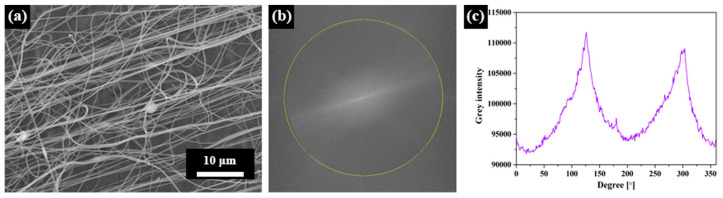
Fast Fourier transform (FFT) conversion from an SEM image to the intensity spectrum: (**a**) SEM image of nanofiber mat; (**b**) FFT frequency spectrogram; (**c**) grey intensity spectrum. From [113], copyright (2020), originally published under a CC-BY license.

Another possibility to evaluate fiber orientation automatically in ImageJ is given by the inbuilt fast Fourier transform (FFT) function as well as the Oval Profile plugin to receive a radial direction intensity plot [110,111,112], as depicted in Figure 6 [113]. The latter can also be given as a polar plot [113], which is often more intuitively understandable.

These automatic orientation examinations have the advantage of taking into account all fiber parts, while manual calculations naturally have to be limited to certain parts of the fibers and are thus susceptible to subjective decisions of the evaluator. On the other hand, automatic calculations of the fiber orientations are highly error-prone if the fibers are too thin, i.e., only a few pixels per diameter, which will lead to favoring 0°, ±45° and ±90° orientations [110]. Thus, the choice of the images will potentially influence the results and has to be done with care.

## 7. Surface Roughness

The surface roughness of electrospun nanofiber mats can influence their hydrophobicity to a certain extent. When researchers mention measuring the roughness related to electrospun nanofiber mats, sometimes the roughness of the whole membrane is meant, while in other cases the roughness of single nanofibers is addressed. Correspondingly, different measurement methods are necessary to detect these different orders of magnitude of roughness.

**Figure 7:**
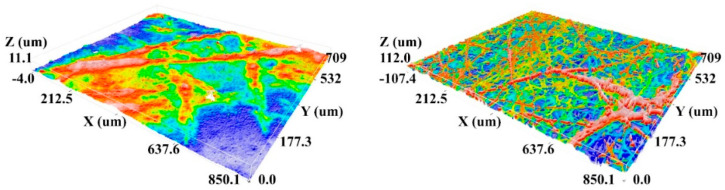
Confocal laser scanning microscopy (CLSM) images of needle-electrospun nanofiber mats from polyurethane (PUR, **left image**) and polyvinyl butyral (PVB, **right image**). From [61], copyright (2020), originally published under a CC-BY license.

Havlícek et al., e.g., show roughness measurements based on confocal laser scanning microscope (CLSM) images [61]. As preparation, they coated the investigated samples with a thin gold layer to enable better visibility of the relatively transparent nanofibers. In this way, 3D maps of the nanofibrous surfaces were prepared, as depicted in Figure 7, from which different roughness parameters could be calculated [61]. As the images show, the resolution of these images is much lower than in SEM images, so that in the lateral direction, only thicker fibers with diameters of some hundred nanometers are visible. This technique is thus only suitable to detect the roughness of a whole nanofiber mat, not of a single nanofiber surface.

If the latter is required, usually SEM or atomic force microscopy (AFM) images are investigated. However, some research groups also investigated the surface roughness of whole nanofiber mats by SEM or AFM.

Field emission SEM (FE-SEM) images were used by Shahverdi et al., who investigated nanofiber mat surfaces with Fiji software (National Institute of Health, 9000 Rockville Pike, Bethesda, MD 20892, USA), leading to relatively noisy 3D images for most samples on which a qualitative comparison of the fiber roughness was performed [114]. El-Morsy et al. used Gwyddion (http://gwyddion.net/ (accessed on 25 March 2023)) to evaluate FE-SEM images, showing average roughness R_a_ of around 100 nm for different fiber material compositions, again with high noise [115]. Other studies show similarly noisy 3D maps, created by Gwyddion or other software from SEM images [116], although the noise could be reduced by using SEM images with higher magnification [117]. Nevertheless, this problem generally occurs during the transfer of SEM images into 3D maps according to the SEM grey scales; more realistic 3D maps need more sophisticated model creation [118].

**Figure 8:**
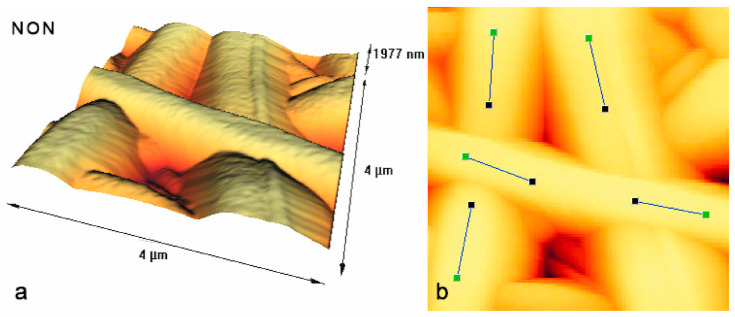
AFM mages of free-surfactant (“non”) electrospun nanofiber mats. (**a**) 3D image and (**b**) 2D image. From [120], copyright (2021), originally published under a CC-BY license.

This is why many groups use AFM measurements instead, which directly measure the fiber heights and where the color code thus directly gives a 3D map of the nanofiber surfaces [119]. Beigmoradi et al. measured the roughness along the fiber axis, as indicated in Figure 8, and found an average roughness R_a_ in the range of 0.5–8 nm for different fibers [120]. As Figure 8 shows, there is no problematic noise that would make the evaluation unreliable. Nevertheless, it is necessary to obtain AFM images with sufficient resolution [121]; otherwise, nanofiber surface evaluation is not possible with AFM, either [122].

Besides the aforementioned methods based on surface images, taken by SEM or AFM, it is also possible to use a laser surface profilometer. In this way, Kichi et al. reported roughness R_a_ in the range of 3–6 µm, i.e., apparently taking into account a larger area of the nanofiber mat, as could be expected due to the optical measurement and the correspondingly limited resolution [123]. Even a mechanical stylus-based profilometer was used to measure the roughness of nanofiber mats, finding R_a_ values around 160–260 nm, however, for a lateral resolution of approx. 60 µm [124].

As this short overview shows, roughness values can be detected with a broad range of different techniques, although it is important to mention which resolution can be expected and whether the measurement was performed over a whole nanofiber mat area or along single nanofibers.

## 8. Nanofiber Mat Thickness

While the macroscopic thickness of a nanofiber mat seems to be simply measurable at first glance, there are, nevertheless, diverse methods with their advantages and disadvantages, sometimes influencing the result by the measurement. One of the problematic methods is using a micrometer caliper since its pressure limitation is usually not sufficient to avoid compression of fine nanofiber mats, similarly to microscopic textile fabrics. Liu et al. tried to compensate for this effect by folding the membranes twice before measuring, i.e., by measuring four layers instead of only one [57], while other groups did not comment on this problem [125]. A typical textile thickness measurement instrument, which has a larger measurement area and causes less pressure on the investigated sample, was applied by Pakolpakcil et al., who used a digital thickness gauge for nonwovens and measured at 10 points on the nanofiber mat [126].

To fully avoid this influence of the measurement on the measured value, some studies used optical methods to investigate the thickness of an electrospun membrane. Ryu et al. applied light transmittance measurements to investigate the sample thickness [127]. For this, they prepared nanofiber mats with different electrospinning times between 15 min and 75 min, measured their light transmittance and the thickness, the latter by cross-sectional microscopic images, and used the Beer–Lambert law correlating both values. This enabled a real-time thickness measurement during electrospinning.

Similarly to the aforementioned roughness measurements, but on larger length scales, Adhikari used a confocal microscope to measure the height of samples from the z-stacks along the sample edges [128]. A profilometer was also used to estimate the depth of a cross-sectional cut by scanning the surface perpendicular to the cut [129]. Naturally, it is also possible to directly investigate the cross-section along a cut through the nanofiber mats by SEM [130,131].

## 9. Hydrophobicity/Hydrophilicity

Hydrophobic or hydrophilic properties of nanofiber mats can be significantly influenced by surface functionalization, e.g., by plasma treatment [132]. The hydrophobic or hydrophilic properties of nanofiber mats are mostly determined by contact angle measurements, mostly applying the sessile-drop method in which a small droplet (e.g., with 5 µL volume [49,133], sometimes less [43]) is placed on the sample, and a microscope with camera is used to take photographs from the side, often at defined times, enabling fitting the contact angles on the photographs. There are also contact measurement instruments that are commercially available [38,42,44,46,48,108,134]. For custom-made setups, evaluation of the contact angles is possible with ImageJ, either manually or with a plugin such as DropSnake [57].

**Figure 9:**
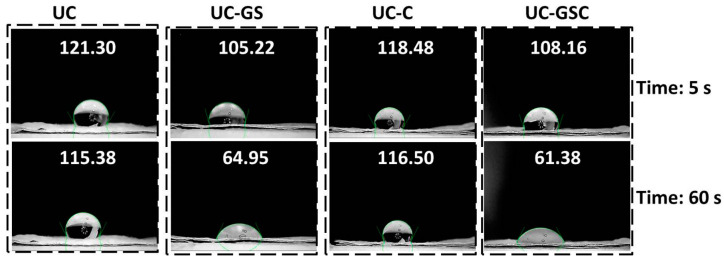
Contact angle measurements of different electrospun nanofiber mats prepared from polyurethane/cellulose acetate, 5 s and 60 s after contact with water droplet. From [44], copyright (2020), with permission from Elsevier.

As an example of the time-dependent change in the hydrophobicity of a nanofiber mat surface, Figure 9 shows measurements taken 5 s and 60 s after the droplet was placed on the membrane, respectively. While most groups used (deionized) water for their contact angle measurements [45,53,70,93,133], others chose PBS solution as the right medium for these examinations [101].

**Figure 10:**
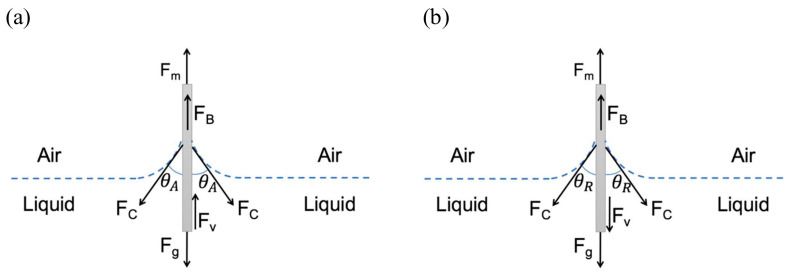
(**a**) Schematic of the advancing motion of the solid plate into the pool of the liquid. (**b**) Schematic of the receding motion of the solid plate out of the pool of the liquid. From [135], copyright (2018), with permission from Elsevier.

An interesting alternative to the contact angle measurements is given by the Wilhelmy plate method, as depicted in Figure 10, for advancing and receding motion [135]. In this method, a plate is immersed into a fluid or retracted from it, allowing measuring the dynamic contact angle. This method was used by Kahdim for contact angle measurements on their nanofibrous scaffolds and showed relatively small standard deviations, i.e., gave reproducible results [39].

Interestingly, other typical textile tests regarding the water repellence of macroscopic textiles, such as the spray test according to AATCC standard test 22 and the water/alcohol solution resistance test according to AATCC 193 and 118 [136,137,138], were not found for electrospun nanofiber mats.

## 10. Water Uptake

The water uptake of an electrospun nanofiber mat can be defined in different ways—by the uptake in the pores around the fibers (cf. Section 2), or by the uptake inside the fibers, causing swelling, which is especially the case for electrospun hydrogels [139]. The water uptake of the material itself can be tested in the bulk form, e.g., by measuring the water uptake of a film of the examined material [38]. It is calculated by

(4) $$Water\;uptake=\frac{m_{1}-m_{0}}{m_{0}}\times 100\%$$

with the masses $m_{0}$ of the dry sample and $m_{1}$ of the sample after immersion in water for a defined time. Often, distilled or deionized water is used, and immersion times are usually around 1–2 days [38,42,140]. The water uptake may vary upon adding fillers, such as nanoclays [138]. While typical values of water uptake are around several percent for many materials, it can also be in the range of 200–600% for very hydrophilic, porous scaffolds [38,43,49,134,141]. For hydrogels, even increasing values of several thousand percent during a few minutes were measured [45,142,143]. Besides water, several papers used PBS solution for fluid uptake tests since cell cultivation often occurs in this medium [38,49,101,134].

## 11. Mechanical Properties

The mechanical properties of electrospun nanofiber mats depend on the fiber material and orientation, but also on the crystallinity of the fibers. They are mostly investigated by tensile tests [56,57], often with test speeds of 1–10 mm/min [41,42,90], sometimes even 20–30 mm/min [46,101], depending on the sample size and elongation at break. A few papers report measuring stress–strain curves with a constant force ramp rate, e.g., 0.3 N/min [88].

Safari et al. examined the difference between dry and wet state and found a significantly higher elongation at break and lower tensile strength in the wet state of Poly(*N*-vinylcaprolactam)/poly(vinyl acetate) copolymer nanofiber mats [38]. Zadeh et al. found that the percentage of carbon nanotubes in the polyurethane nanofiber mats influenced Young’s modulus of the investigated samples [49]. Zhang et al. reported high tensile strength and modulus for PLA, high elongation at break for PCL, and averaged values for PLA/PCL blends [70]. Bazzi et al. found a significant increase in Young’s modulus and toughness by adding a small amount of graphene nano-platelets to chitosan/polyvinyl alcohol electrospun nanofiber mats [68].

A very special tensile test, based on single electrospun fibers, was reported by Munawar and Schubert [144]. Working with well-aligned fibers, they rolled a fiber bundle for the tensile testing, clamped it in a single-fiber tensile tester, and afterwards cut the tested area and weighed the tested part of the fiber bundle to enable calculation of Young’s modulus. In this way, they could measure along the fiber axes instead of taking the mechanical properties of a whole nanofiber mat, averaging over arbitrary fiber orientation.

**Figure 11:**
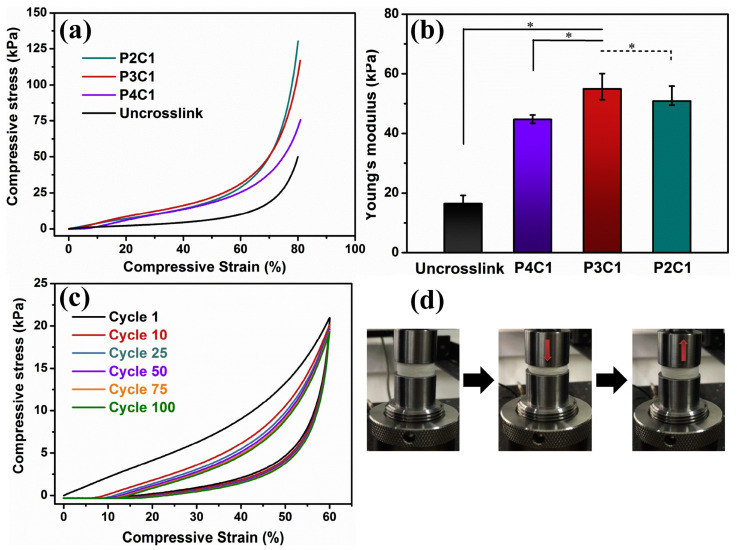
Mechanical tests on scaffolds from poly(lactic acid)/regenerated cellulose/citric acid in a ratio of x:1:1, named PxC1, and un-crosslinked without citric acid. (**a**) Compressive stress–strain curves and (**b**) Young’s modulus of the different scaffolds, * indicating significant differences; (**c**) 100 cyclic compressive fatigue tests of P3C1 scaffolds under a compressive strain of 60%; (**d**) photographs of the P3C1 scaffolds under a compressing and releasing cycle. From [45], copyright (2020), with permission from Elsevier.

Besides these typical textile tests, some authors also report compressive tests of their samples. This was the case for 3D specimens, e.g., prepared by combining electrospinning and freeze-drying [43,45,133]. Chen et al. investigated cyclic compressive stress–strain curves on their 3D scaffolds, showing the usual hysteresis loops, as depicted in Figure 11 [45]. Chen, on the other hand, found super-elastic and shape-recovery properties and reported enhanced elastic modulus and reduced energy loss during cyclic testing with a gelatin coating [133].

Besides tensile and compressive tests, some groups also reported bursting tests of nanofiber mats. Jalalah et al. applied the standard bursting strength test according to ISO 13938-2:1999 and found a linear correlation between nanofiber mat thickness and bursting strength [89]. Nejad et al. found a significant increase in the bursting strength of their nanofiber mats by adding PCL to poly(ethylene terephthalate) (PET). It was, however, calculated from the tensile strength tests [53]. The latter also tested suture retention according to ISO 7198:2016 by tensile tests with a well-defined suture thread that was inserted 2 mm from the top edge of the electrospun strip, so that the tensile test led to pulling the suture through the graft [53].

As these examples show, tensile tests are not the only tests possible to be performed on electrospun nanofiber mats; however, the most meaningful tests should be chosen for the planned application.

## 12. Electrical Conductivity

The conductivity of nanofibers depends on their material, thickness, crystallinity, etc. Conductive nanofiber mats can stimulate cell attachment, proliferation and differentiation [145]. This is why the conductivity of electrospun membranes is often measured [9]. On the other hand, soft and compressible textile fabrics generally pose a challenge to measurements of their conductivity since the contact between the measuring instrument and conductive parts of the sample may be prohibited by non-conductive fibers, and the fibrous structure reduces the contact area if contact pins are used, as is usual for multimeters [146]. Generally, samples can be measured with the two-electrode methods (as in common multimeters), the four-electrode method, which is capable of eliminating the contact resistance, and methods with even more electrodes [147].

The four-wire measurement (also known as four-terminal sensing or four-point probe) uses two outer current-introducing and two inner voltage-sensing electrodes, in this way becoming independent from the contact resistances. The van der Pauw method works similarly: while the four contacts are not aligned but positioned along the sample perimeter, the van der Pauw method measures the average sample resistivity, whereas the linear four-point probe method measures the resistivity along the electrode orientation [148]. Due to the expected high contact resistance, textile fabrics should normally be measured with the linear four-point probe or the van der Pauw method, depending on the desired information and the sample geometry.

Nevertheless, multimeters are often used to investigate the resistance of electrospun nanofiber mats, either with constant voltage [133] or by measuring the voltage-dependent current [68]. Zadeh et al. used a special four-probe cell to measure the sample impedance in a frequency range from 1 Hz to 100 kHz and calculated the sample resistance from this curve [49], while Zarei et al. [130] as well as Simsek et al. [149] directly measured the resistance using a four-probe method.

Munawar et al. decided to use another solution for the potentially high contact resistance—they coated the ends of the measured nanofiber bundles with silver ink to increase the conductivity of the contacts, so that they could perform reliable two-point resistance measurements [144].

Finally, it should be mentioned that in spite of the importance of the conductivity of electrospun scaffolds, most papers only report on conductivity measurements performed on the spinning solutions, since this parameter directly influences the electrospinning process.

## 13. Water Vapor Permeability

The water vapor permeability of an electrospun membrane is correlated with its porosity and is especially important for wound dressing applications, where too high water vapor permeability results in fast hydration and thus scars, while too low values let exudates accumulate and thus increase the risk of infection [150,151]. Quantitatively, the water vapor transmission should be between 76 and 9360 g/(m^2^ day) to improve wound healing [152,153]. Gu et al. reduced this optimum window to 2000–2500 g/(m^2^ day) [154].

Mostly, the water vapor transmittance is measured by gravimetry, where the sample is fixed on the opening, with defined diameter (e.g., 1.23 cm), of a round bottle that is filled with a defined volume of distilled water (e.g., 5 mL) and placed in an oven at typically 37 °C for 24 h [155]. The water vapor transmittance (WVTR) is calculated as

(5) $$\mathrm{WVTR}=\frac{-\Delta W}{AT}$$

with the mass change −ΔW of the water in the container, the exposed area A and the measurement time T [42]. Usually, the same measurement is performed with an open container as the reference [155].

Chaiarwut et al. found a WVTR of 9335 g/(m^2^ day) for the positive control (open bottle), i.e., the upper border of the desired water vapor transmission to improve wound healing, and values around 2500 g/(m^2^ day) for different nanofiber mats from PCL [101]. Samadian et al. reported a similar value for cellulose acetate/gelatin nanofibers [156], as did Zheng et al. for crosslinked pectin nanofiber mats [157]. Slightly higher values of around 3500 g/(m^2^ day) were found for polyamide-6/polyvinylpyrrolidone nanofibers [48], while Esmaeili reported smaller values of around 1300 g/(m^2^ day) [44]. Salemi et al. used a test temperature of only 33 °C, resulting in lower reference values of around 7440 g/(m^2^ day) and around 3600 g/(m^2^ day) for their poly (caprolactone)/poly (vinyl alcohol)/collagen nanofiber mats [158].

While this test method, also known as the cup test [159], is most often used to investigate the water vapor permeability of electrospun nanofiber mats, it is nevertheless not free of potential errors. As Mustapha et al. discussed, the measured water vapor permeability actually consists of three different resistances: those of the air cavity over the water, then the actual intrinsic membrane resistance, and finally the boundary air layer resistance, the latter of which the authors suggested to reduce by introducing a fan blowing the air above the cups away, while they used an open control cup to measure the air resistance by comparing the evaporation from this cup with an analytical model [160]. Slightly different methods are described in ASTM E96-95, where the cup is placed in a desiccator containing saturated MgCl_2_ or Mg(NO_3_)_2_·6H_2_O solution to provide a constant relative humidity, and the water transferred through the film is measured by an analytical balance [161,162]. Other tests, such as the sweating guarded hot plate test according to ISO 11092, the inverted cup method, the dynamic moisture permeation cell test method, the desiccant inverted cup test method, etc., are only scarcely reported for nanofiber mats in the literature [163]. Nevertheless, it is important to mention the method used, since even the methods giving results in the same units (typically g/(m^2^ day)) deliver quite different results for identical samples [164].

## 14. Air Permeability

Air permeability is one of the parameters often measured for macroscopic textiles, but less often for electrospun nanofiber mats, potentially because it can partly be estimated from water vapor transmission tests [153]. Nevertheless, some studies report measuring the air permeability of nanofibrous scaffolds directly, usually giving the transmitted air volume per area and time, i.e., in the unit cm³/(cm^2^ s) or cm/s. Pakolpakcil et al. used a commercial air permeability tester at a fixed pressure of 100 Pa and a test area of 20 cm^2^, resulting in values of around 10–12 cm/s [126]. Using the same parameters, Sun et al. found values of around 1–3 cm/s for their nanofiber mats electrospun from polyamide and multi-wall carbon nanotubes [165]. Yardimci et al. combined the same pressure with a test area of 50 cm^2^ and found values of around 2.7–2.9 cm/s [166]. Slightly different parameters of 125 Pa and 38.3 cm^2^ were used by Kim et al., who measured air permeability values in the range of 3.6–5.3 cm/s for their polyurethane-coated nanofiber mats [167]. Sarwar et al., on the other hand, applied a constant air flow of 2 cm/s and measured the air permeation in liters per minute [168].

Other methods, such as methods based on falling pistons in a chamber closed by the investigated textile fabric [169,170,171,172,173], are usually not reported for nanofiber mats.

## 15. Thermal Properties

The thermal conductivity of an electrospun nanofiber mat is often correlated with its electrical conductivity; however, in many cases, only the thermal conductivity is measured. Depending on the planned application, sometimes a high thermal conductivity is sought, while often the high porosity of a nanofiber mat, combined with the low thermal conductivity of most polymers, is used to prepare heat-blocking nanofibrous membranes instead.

Thermal conductivity can be evaluated, e.g., by a diffusivity measurement instrument at a defined temperature, often not far above the room temperature [174]. Measurements at high temperatures, however, are also possible using the hot disk method, e.g., using infrared thermography on the upper side of the sample, which is placed on the hot disk [175,176,177]. Besides a hot plate to heat up one sample side, a light flash, e.g., from a pulsed xenon lamp, can also be applied to heat one side of the sample [178,179,180].

## 16. Conclusions

The physical parameters that are typically measured on electrospun nanofiber mats for biotechnological applications can be described as morphology-related ones (porosity, pore size and specific surface area, fiber diameter and orientation, roughness and thickness of the nanofiber mat), hydrophobic/hydrophilic properties and water uptake, mechanical properties, and electric conductivity as well as water vapor and air permeability. Other potentially interesting properties, such as solute transport, which could be measured in a side-by-side diffusion chamber [57], are only scarcely reported. Table 1 gives a brief overview of these properties and typical measurement procedures as well as sample dimensions and test standards, if mentioned in the papers.

Since most of these parameters can be measured in different ways, it is generally highly recommended to precisely define the measurement technique, regarding the physical principle, environmental conditions and all parameters that can be modified. This should also become a standard when commercial instruments are used but whose exact function is often not completely known even to the user and cannot be reproduced by other researchers who do not own the same instrument, or for country-based standards that are not necessarily available worldwide. Generally, in many cases, more exact descriptions or definitions of the measured parameters are necessary, e.g., regarding the term “roughness”, which can mean the surface roughness of a single nanofiber, but also the areal roughness of a whole nanofiber mat.

Moreover, some of the typical textile test methods, such as the simple spray test or water/alcohol test for the determination of the hydrophobicity of a fabric, or tests well-known from other research areas, such as thermoporometry, should be investigated regarding their usability for electrospun nanofiber mats, and their limits should be discussed as well as the possibilities they offer by enabling more tests if highly specialized equipment to measure a certain parameter is not available.

We hope that this review will encourage colleagues to test some new measurement techniques and extend their experimental descriptions so that all experiments can be reproduced by other research groups.

## Figures and Tables

**Table 1:** Physical properties, typical measurements, sample dimensions and standards.

Physical Property	Test Procedures	Dimensions, Standards	References
Porosity	Fluid uptake		[39,40,41,42,43,44,45]
	Gas pycnometer	ASTM D2000	[47]
	Apparent density		[57]
Pore size distribution	SEM images, ImageJ		[52,61]
	Thermoporometry		[85,86,87]
Specific surface area	BET isotherms		[88,89,90]
Nanofiber diameter	SEM images, DiameterJ		[51,60]
Nanofiber orientation	SEM images, ImageJ		[54]
Surface roughness	CLSM		[61]
	SEM and Fiji software		[114]
	SEM and Gwyddion software		[115,116]
	Atomic force microscopy		[119,120,121]
Nanofiber mat thickness	Textile thickness tester		[52]
	Laser profilometer		[54]
	Micrometer caliper		[57]
Hydrophobicity	Sessile drop		[43,44,50,53,88]
Water uptake	Mass difference dry/wet		[140,141,142,143]
Mechanical properties	Tensile tests	10 mm × 10 mm	[131]
		15 mm × 20 mm	[45]
		Length 100 mm, ASTM D882	[46]
		10 mm × 30 mm, ASTM D882	[48]
		10″ × 3″, EN ISO 13934:1:1999	[89]
Electrical conductivity	Impedance measurement		[88]
	Conductivity meter		[114]
Water vapor permeability	Bottle permeation test	1.18 cm^2^	[42]
		1.77 cm^2^	[44]
Air permeability	Air permeability tester	20 cm^2^	[126]
		50 cm^2^	[166]
		38.3 cm^3^	[167]
Thermal conductivity	Hot plate		[175,176,177]
	Light flash		[178,179,180]

## Data Availability

No data were produced in this review paper.
